# Evaluation of Wild Potato Germplasm for Tuber Starch Content and Nitrogen Utilization Efficiency

**DOI:** 10.3390/plants9070833

**Published:** 2020-07-02

**Authors:** Silvia Bachmann-Pfabe, Klaus J. Dehmer

**Affiliations:** Leibniz Institute of Plant Genetics and Crop Plant Research (IPK), Genebank Department, Satellite Collections North, Gross Luesewitz Potato Collections, Parkweg 3a, 18190 Gross Luesewitz, Germany; dehmer@ipk-gatersleben.de

**Keywords:** genetic resources, *Solanum chacoense*, stress tolerance

## Abstract

Potato wild relatives provide a considerable source of variation for important traits in cultivated potato (*Solanum tuberosum* L.) breeding. This study evaluates the variation of tuber starch content and nitrogen utilization efficiency (NutE) in wild potato germplasm. For the experiments regarding starch content, 28 accessions of ten different tuber-bearing wild *Solanum*-species were chosen, and in vitro plantlets were raised from seeds. Twenty plantlets (= genotypes) per accession were then cultivated in the greenhouse until natural senescence and tuber starch content was determined. The average tuber starch content across all genotypes tested was 21.7% of fresh mass. Contents above 28% of fresh mass were found in 50 genotypes, belonging to the species *S. chacoense*, *S. commersonii*, *S. jamesii*, and *S. pinnatisectum*. Subsequently, 22 wild genotypes revealing high tuber starch contents and four modern varieties of cultivated potato were studied as in vitro plantlets under optimal and low N supply (30 and 7.5 mmol L^−1^ N). Low N supply lead to a genotype-dependent reduction of shoot dry mass between 13 and 46%. The majority of the wild types also reduced root dry mass by 26 to 62%, while others maintained root growth and even exceeded the NutE of the varieties under low N supply. Thus, wild potato germplasm appears superior to cultivars in terms of tuber starch contents and N utilization efficiency, which should be investigated in further studies.

## 1. Introduction

Besides being one of the most important food crops worldwide, potato (*Solanum tuberosum* L.) plays an important role in industry due to its starchy tubers. Starch is used in bakery products, thickening products, soups and noodles but also for the production of paper, textiles, building materials, pharmaceutical products, chemicals and biodegradable packaging materials [1]. Compared to other starches, potato starch has superior characteristics because it is easily isolated, of high purity and of large granule size, needs low temperatures for gelatinization and produces gels with highest viscosity [2]. Depending on genotype and growing environment, a fresh potato tuber contains about 20% of dry mass, 60–80% of which is starch [3]. Dedicated starch varieties may even reach a starch content of up to 23% of fresh mass [4]. The nitrogen (N) fertilizer regime influences starch yield by positively affecting canopy development and photosynthesis efficiency, dry matter partitioning to the tubers, tuber bulking and tuber yield formation [5,6]. Furthermore, field N availability influences starch quality parameters, such as granule size, viscosity and breakdown [7]. Consistently, Maltas et al. [8] reported a highly significant effect of different N fertilizer rates on total tuber yield, the percentage of large tubers and starch concentration under field conditions in cv. Bintje and Laura.

Depending on environmental conditions and genotype, the potato crop has been found to remove 90 to 190 kg N ha^−1^ [9]. However, the shallow, less branched and less dense root system of potato does not allow the exploration of a large soil volume or to retrieve nitrogen from deeper soil layers, and hence, potato demands a high level of readily available soil N at the right period of growth [5,9,10,11,12]. In a review, Iwama [13] reported that most of the potato roots are present in the upper 30 cm of the soil and only a small fraction extends to 100 cm. In combination with the fact that potatoes are often cultivated on coarse and sandy soils and under irrigation, these areas face an increasing potential for nitrate leaching and contamination of groundwater [14].

Thus, improving the N use efficiency in potato production is not only of economic, but also of environmental concern, and different measures such as split application of N or foliar application of urea are being discussed in order to reduce N leaching [5]. Improving the N uptake and use efficiency of the potato crop itself is also an important approach, and many studies evaluated the N use efficiency in cultivated potatoes [8,15,16,17,18,19,20,21]. In contrast, only few studies evaluated the N use efficiency and/or tuber starch contents of native Andean cultivars or wild potato germplasm [22,23,24,25]. The wild relatives of the cultivated potato could be an important source of variation for root length and morphology, tuber starch content and N use efficiency. The secondary and tertiary genepool of potato has intensively been studied as a source of disease resistance [26,27,28], and was used, amongst others, to improve foliar late blight and nematode resistance of *S. tuberosum* [29,30]. In terms of N use efficiency, Errebhi et al. [22] compared 39 wild potato accessions of 23 species with three cultivated varieties under high and zero N in the field. They found some wild potato genotypes which were able to take up significantly more applied N than their cultivated relatives. Genotypes of *S. microdontum* and *S. chacoense* were identified as the ones with the highest N uptake efficiency (NupE) [22]. Selected native Andean cultivars indicated a similar nitrogen use efficiency to commercial cultivars, but showed, despite different environmental conditions, a highly consistent performance across a two-year field study [24]. To our knowledge, the most comprehensive study of tuber starch content and quality in exotic germplasm, was provided by Jansen et al. [25]. Accessions of 46 wild and cultivated potato species showed a high variation in starch contents ranging between 3.8 and 39.6% of fresh mass. Highest starch contents were predominately found in genotypes of species *S. pinnatisectum* and *S. chacoense* [25]. 

Based on the above-mentioned findings, our study aimed to (I) update and assess the variability of the tuber starch contents in wild potato germplasm and to (II) study the nitrogen use efficiency of genotypes with high tuber starch contents in relation to modern cultivars.

## 2. Results

### 2.1. Variation of Tuber Starch Contents in Wild Potato Germplasm after Greenhouse Cultivation

In 2013, altogether 28 different wild potato accessions (= populations) representing ten different species were cultivated in the greenhouse to evaluate their tuber starch contents. For each accession 20 different genotypes were cultivated as in vitro plantlets, however, the results only include genotypes which produced sufficient tubers for starch analysis (in total 506 genotypes, Table 1). On average, of all the 506 genotypes analyzed, the starch content in the tubers amounted to 21.7% of fresh mass (FM). The lowest average starch content with 14.2% of FM was measured for accession Gross Luesewitz Potato Collections (GLKS) 31559 (*S. stenotomum*), while accession GLKS 30211 (*S. commersonii*) showed the highest starch content with on average 30.0% of FM. Interestingly, all genotypes of accession GLKS 30211 showed high tuber starch contents ranging between 26.4 and 33.3% of FM, indicating a rather low variation within this population (Table 1). From *S. chacoense,* all the 15 accessions had an average starch content of 22.5% of FM and showed a rather low variation between the populations (CV = 8.71%), and a higher within population variation (CV: 11.5–23.9%, Table 1). Ten accessions of *S. chaoense* encompassed genotypes with a tuber starch content higher than 28% of FM, a target value which was considered as selection criterion for high-starch genotypes in this study. Regarding *S. pinnatisectum*, the tubers of the five accessions studied, had an average starch content of 22.2% of FM, but showed starch contents up to 36.6% of FM. Altogether 11 genotypes of three accessions produced starch contents higher than 28% (Table 1). 

For subsequent N efficiency experiments, genotypes with starch contents higher than 28% of FM were to be used. In *S. chacoense*, altogether 23 genotypes were identified revealing a tuber starch content above 28% of FM. Overall, 15 genotypes, belonging to accessions GLKS 30135, GLKS 30154, GLKS 30156, GLKS 30159, GLKS 30160, GLKS 30177, GLKS 30181, GLKS 30916, and GLKS 30995 were selected for the N experiments and re-cultivated in 2014 to validate their starch contents (Table 2). In *S. pinnatisectum*, 11 genotypes showed tuber starch contents above 28% of FM, and genotype GLKS 31600_10 with a starch content of 36.6% was selected for up-coming experiments and re-tested in 2014. Additionally, two genotypes with the highest starch contents of *S. microdontum* (GLKS 30688_04, GLKS 30688_12) and *S. stenotomum* (GLKS 31559_11, GLKS 31559_14) as well as one genotype of *S. tuberosum* subsp. *andigena* (GLKS 34995_18) were selected for the N efficiency experiments in order to cover a broader spectrum of *Solanum* species, even if they had starch contents below 28% of FM (Table 2). The tuber starch contents determined in 2013 and after re-testing of selected genotypes in 2014 correlated well (r = 0.72, *p* ≤ 0.01), confirming the high-starch properties of the majority of the selected accessions.

### 2.2. Dry Yield of Shoots, Roots and Root-DM:Shoot-DM Ratio in the N Experiments

Shoot and root DM as well as the root-DM:shoot-DM ratio were predominately affected by the genotype and, to a lesser extent, by the factor treatment. For these traits, the factor genotype explained up to 68% of the variation in the data, while the factor treatment explained between 6 and 22%. The genotype × treatment interaction explained 11 and 15% of the total variation for root DM and root-DM:shoot-DM ratio, respectively, but played only a minor role for shoot DM (3.58%, Table 3).

In the high N (30 mmol L^−1^) treatment, the shoot DM of the genotypes varied between 214 and 682 mg vessel^−1^ (Table 4). The lowest shoot DM was observed for the genotypes GLKS 31600_10, GLKS 30177_17 and cv. Kiebitz, while the highest biomass was achieved by the genotypes GLKS 30177_20, GLKS 30181_06 and GLKS 30160_15. These genotypes even exceeded the performance of cv. Tomba, which showed the highest shoot DM amongst the standard varieties. Under low N supply (7.5 mmol L^−1^), shoot DM ranged between 156 and 549 mg vessel^−1^. Shoot DM decreased under low N supply on average by 115 mg vessel^−1^ (23%) and the shoot biomass reduction was significant for all the genotypes tested, except for cv. Kiebitz and cv. Eurobravo (Table 4). The strongest reduction (>30%) was observed for genotypes GLKS 30135_19, GLKS 30995_18, GLKS 30177_02 and GLKS 31559_11. A moderate shoot DM reduction (15 to 20%) at simultaneously high yields in the control was observed for genotypes GLKS 30135_05, GLKS 30160_13 and GLKS 30177_20. Again, cv. Tomba produced the highest shoot DM amongst the standard varieties under low N supply. However, several wild potato genotypes performed as well or even exceeded the shoot DM of cv. Tomba under reduced N conditions (Table 4).

The root DM varied between 30 and 337 mg vessel^−1^ in the control treatment. The lowest root growth (<80 mg vessel^−1^) was observed for genotypes GLKS 31600_10, cv. Kiebitz, GLKS 30181_18 and GLKS 31559_14, while the genotypes GLKS 30154_09, GLKS 30160_13 and GLKS 30177_20 produced more than 300 mg vessel^−1^. Under low N conditions, the root DM varied between 41 and 219 mg vessel^−1^. The different genotypes either maintained or decreased root DM due to N deficit. The decrease was highest (about 60%) for the genotypes GLKS 30135_05, GLKS 30177_02, GLKS 30181_06, while the genotypes GLKS 30177_15, GLKS 30181_18, GLKS 30688_04, GLKS 30688_12, GLKS 31559_14, GLKS 31600_10 and cv. Kiebitz maintained root mass. Most interestingly, genotype GLKS 30177_15 produced a high root mass in the control and maintained it under reduced N conditions. Amongst the cultivars, cv. Tomba produced the highest root mass in both treatments. However, several wild potato genotypes produced a significantly higher root biomass under control (GLKS 30160_13, GLKS 30177_20) or low N conditions (GLKS 30160_15, GLKS 30177_15, GLKS 30177_20) compared to cv. Tomba (Table 4).

Relating the root to the shoot biomass improves the understanding of genotype-specific reactions to low N supply. The root-DM:shoot-DM ratio varied strongly between the wild types, ranging from 0.14 to 0.61 in the control and 0.10 to 0.48 in the low N treatment, but was generally comparable to that of the standard varieties (Table 5). High values above 0.50 were calculated for genotypes GLKS 30154_09, GLKS 30177_01, GLKS 30160_13 and GLKS 30177_17, indicating a strong root growth in relation to the shoot. Genotypes reacted differently to low N supply, either by maintaining (13), reducing (11) or increasing (2) root-DM:shoot-DM ratio. The genotypes GLKS_31559_14 and GLKS_31600_10 showed the lowest root-DM:shoot-DM ratio in the control, but increased root growth at the expense of the shoot under low N.

### 2.3. N Uptake, N Partitioning and N Efficiency Parameters in the N Experiments

Interestingly, the genotypic variation for the shoot and total N uptake was low and explained only about 4% of the total variation. In contrast, the factor genotype explained between 20 and 60% of the total variation for the traits root N uptake, NutE and N partitioning (root-N:shoot-N ratio). Additionally, a clear genotype × treatment interaction was observed for the root-N:shoot-N ratio (Table 3). In the control treatment, the average shoot N uptake was 22.0 mg N vessel^−1^ and varied only by ± 3.18 mg vessel^−1^ (CV = 14.5%). In the low N treatment, shoot N uptake was on average reduced by 15.3 mg N vessel^−1^ (69%), and the reduction was significant for all the genotypes tested (Table 4). Amongst the standard varieties, cv. Tomba had the highest N uptake in the control, and many wild potato genotypes achieved shoot N uptakes as high as cv. Tomba. However, under low N supply, the wild potato genotypes generally lag behind cv. Tomba, except for genotype GLKS 30135_05. 

In both treatments, root N uptake accounted for approximately one quarter of the shoot N uptake but showed a stronger variation between the genotypes. In the control, root N uptake was on average 4.59 mg vessel^−1^ and varied by ± 1.36 mg vessel^−1^ (CV = 29.6%). Under low N supply, root N uptake decreased on average by 59% to 1.88 mg vessel^−1^. The reduction was significant for all the genotypes tested, except for GLKS 31600_10. Again, cv. Tomba was the best cultivar in terms of root N uptake in both treatments, but, in contrast to shoot N uptake, root N uptake of many wild potato genotypes even exceed that of cv. Tomba under control as well as under reduced N conditions (Table 4).

**Figure 1:**
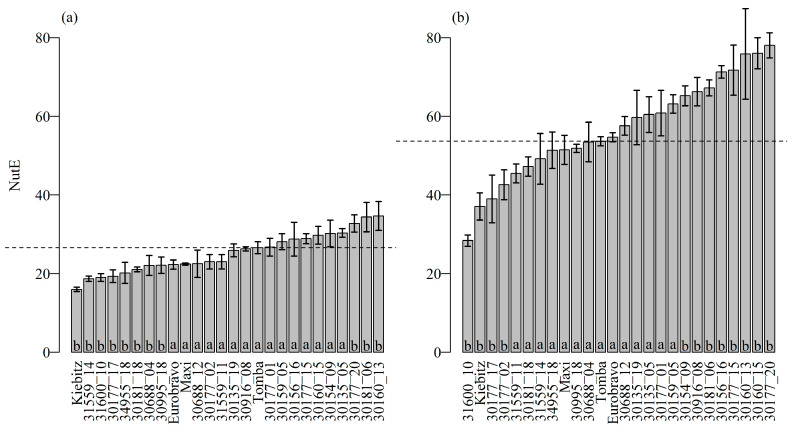
Mean nitrogen utilization efficiency (NutE) of 22 different wild potato genotypes and the four standard varieties under optimal (**a**) and reduced N conditions (**b**) after 21 days of cultivation in a climate chamber. Error bars indicate standard deviation, the dotted line indicates the mean NutE of the best cv. Tomba. The different letters at the bottom of the bars indicate whether there is a significant difference to the best cultivar Tomba (“b”) or not (“a”, Dunnett’s test *p* ≤ 0.05).

Total N uptake (shoot + root N uptake) generally ranged from 22.1 to 31.4 mg vessel^−1^ (except GLKS 31600_10 with only 12.8) in the control, and from 6.90 to 11.3 mg vessel^−1^ in the reduced N treatment. Relating the total N uptake of the plant to the total amount of N supplied revealed an average NupE of 102% in the control and 132% in the low N treatment. This indicates that the plants took up all the N provided via the nutrient solution and that an additional amount of N was introduced into the system via the ten shoot tips. The NutE, expressed as the amount of total biomass (shoot + root dry mass) produced per unit N taken up, increased, on average, from 25 units in the control to 57 units in the low N treatment. NutE differed moderately between the genotypes in the control treatment where it ranged from 16 to 35 units. In the reduced N treatment, NutE varied strongly from 28 to 78 units. Although all genotypes significantly increased their NutE under low N conditions, many wild potato genotypes exceeded the NutE of the standard varieties. For example, in the low N treatment 13 of the wild potato genotypes achieved a similar and eight genotypes a significantly better NutE than the best cultivar Tomba (Figure 1). The N partitioning, as the ratio of N taken up by the root and N taken up by the shoots (root-N:shoot-N ratio), gives insight into the distribution of N within the plant. In the control, most of the wild types showed values similar to that of the standard varieties, while nine wild types showed significantly higher values than cv. Tomba. Under reduced N conditions, the majority of the wild types increased the root-N:shoot-N ratio, while it remained constant for all the standard varieties. This indicates a stronger partitioning of N to the root for wild potato genotypes, in particular under N deficit.

**Figure 2:**
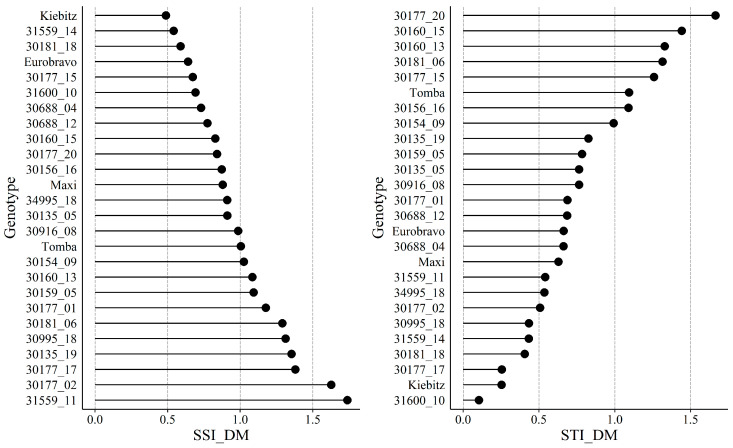
Stress susceptibility index (SSI) and stress tolerance index (STI) of different wild potato genotypes and four cultivars based on their total dry mass production (DM) under optimal and reduced N supply during 21 days of cultivation in a climate chamber. Genotypes are ranked according to their performance, with the best being on top of the graph.

To evaluate the stress performance of the different genotypes, two commonly used indices (stress susceptibility index SSI, stress tolerance index STI) were applied based on total plant dry mass (shoot DM + root DM). In Figure 2 the accessions and genotypes are ranked according to their SSI and their STI from the best to the weakest genotype. The SSI, as a measure of the yield stability under stress conditions, identified cv. Kiebitz, GLKS 31559_14 and GLKS 30181_18 as the three most stable genotypes across both environments, while the reaction to N stress was most pronounced for GLKS 30177_17, GLKS 30177_02 and GLKS 31559_11. On the other hand, the STI identified GLKS 30177_20, GLKS 30160_15 and GLKS 30160_13 as the most promising genotypes, because they produced high total yields under control as well as under stress conditions, whereas cv. Kiebitz and GLKS 31600_10 ranked least, due to relatively low yields in both treatments (Figure 2).

## 3. Discussion

We assessed the tuber starch content in wild potato accessions and studied the reaction of selected wild types to N deficiency. After greenhouse cultivation, the average starch content of all tested wild potato genotypes amounted to 21.7% of FM, but ranged from minimal 7.1 to maximal 36% of FM. This indicates considerable variation for this trait in the wild relatives of cultivated potato. Furthermore, in 50 out of the 506 genotypes, tuber starch content was higher than 28%, which clearly exceeds the starch contents of modern cultivars. Based on our study, germplasm with high tuber starch content is predominately found in *S. chacoense*, *S. commersonii*, *S. jamesii* and *S. pinnatisectum*. Our results are in line with Jansen et al. [25], where tuber starch contents ranged between 3.8 and 39.6% of FM in wild species cultivated in the greenhouse. Here, genotypes of the species *S. chacoense* and *S. pinnatisectum* revealed the highest starch contents. In comparison, 14 modern starch potato varieties cultivated in pot experiments revealed starch contents between 13.9 and 21.9% of FM [20]. Under field conditions, tuber starch contents ranged from 7 to 20% of FM in a set of 300 potato cultivars, breeding clones, landraces and diploid clones [31]. This points out the potential of wild potato germplasm to increase tuber starch contents in cultivars. However, next to the tuber starch content, also the starch yield, as a result of starch content multiplied by tuber yield, plays an important role for industrial starch production. Whether the high starch contents in the wild species will be maintained when tuber size increases due to breeding, has still to be examined. Studies of Schönhals [31] showed that tuber yield is negatively correlated with tuber starch content, while no significant correlation between tuber yield and starch content was found by Bombik et al. [32]. 

An increase in tuber starch content and starch yield should be linked with a high resource use efficiency. This holds particularly true for the element N, because potato cultivation bears high risks of N leaching due to its high demand of readily available N in soil and the small root system of the crop [5,6,14]. Based on our results mentioned above, 22 wild potato genotypes with high tuber starch contents and four commercial cultivars were studied for their N use efficiency by cultivating them as in vitro plantlets in 500 mL vessels filled with a nutrient solution containing 30 or 7.5 mmol L^−1^ N for 21 days in a climate chamber. This system allowed us to screen a high number of plants under low space requirement and highly controlled conditions. Several other reports underline the potential of in vitro cultures for the evaluation of potato germplasm with respect to abiotic and biotic stress or rooting characteristics, because it provides conditions independent from weather conditions, pathogens, N leaching or immobilization events [16,33,34,35]. Our results revealed a high variation in shoot and root DM development between the wild potato genotypes. N deficiency significantly reduced shoot DM for all wild types and the cultivars (except for cv. Kiebitz and cv. Eurobravo), with the extent of shoot DM reduction being genotype dependent. Most interestingly, genotypes GLKS 30135_05, GLKS_30160_13 and GLKS 30177_20 of *S. chacoense* showed a moderate shoot DM reduction due to low N supply whilst producing a comparably high shoot biomass under high N conditions. The root DM varied considerably and the wild types GLKS 30160_13, GLKS 30160_15, GLKS 30177_15 and GLKS 30177_20 clearly exceeded the root growth of the best cultivar Tomba under high and/or low N supply, indicating that wild potato germplasm could considerably contribute to enhance root growth of *S. tuberosum* cultivars. Under N deficit, the genotypes followed different strategies in terms of root development. A significant reduction of root DM was observed for the majority of the genotypes, while seven genotypes maintained root biomass. This was also reflected in the root-DM:shoot-DM ratio which was either maintained (13 genotypes), reduced (11 genotypes) or increased (two genotypes). To sustain or even increase root biomass at the expense of the shoots is a well-known reaction of plants to nutrient deficiency and helps to maintain the nutrient uptake from soil or nutrient solution by exploring a larger (soil) volume [36,37]. In contrast, other wild types seem to preferably invest into shoot growth, probably in order to maintain photosynthetic activity. Different strategies to cope with low N as observed in our experiment are also known from cultivated potatoes. A higher root:total mass ratio under N deficiency was reported for the majority of 17 modern starch and table potato varieties during the course of 18 days of in vitro cultivation [16]. On the other hand, the authors also identified some genotypes which missed the ability to stimulate root growth at the expense of the shoots under N deficiency. This is in accordance with previous studies under climate chamber conditions where some cultivars reduced root FM with increasing N stress, while others showed an increased root development upon N reduction and even maintained root growth at very low N levels [17]. From their studies, Schum et al. [16,17] observed that genotypes with high biomass production and fast nitrogen uptake under high N supply did not enhance root growth under low N and clearly reduced biomass production. On the other hand, genotypes with comparatively slow growth under high N supply increased root mass under low N supply [16,17]. Besides, it should be considered that other well-known responses of plants to nutrient deficiency stress such as changes in the root architecture, increase of root length, root surface area, root volume or number of root hairs [33], is not necessarily reflected in changes of the total root biomass as measured in our experiment. In relation to the total N applied, the plants took up almost all the N available in both treatments (except for GLKS 31600_10). This explains the low genotypic variation for total N uptake (Table 3). In some cases, N uptake of the genotypes even exceeded the amount of N provided via the nutrient solution, probably due to the additional N introduced into the system via the transferred shoot tips. Therefore, it is difficult to evaluate the NupE of the different accessions. Nevertheless, on the basis of the uniform N uptakes of the genotypes, the results give insight into genotype-dependent N partitioning and provide a clear picture in terms of NutE.

Most of the N taken up by the different genotypes was partitioned to the shoots under high N supply in our study. Under low N supply, a clear shift towards the roots was observed for many genotypes (Table 5). This confirms previous results, where generally a higher percentage of N was translocated to the roots or tubers under nutrient deficiency [6,36]. The increase in root-N:shoot-N ratio became especially evident for many wild potato genotypes, while this was less pronounced for the standard varieties.

NutE was on average 25.3 units in the control and increased to 57.3 units under N deficiency and the genotypic variation in NutE was particularly high in the low N treatment (Figure 1). This is in line with several studies [16,17,20,24]. In our experiment, many wild potato genotypes exceeded the NutE of the standard varieties under low N supply. Outstanding genotypes were GLKS 30154_09, GLKS 30916_08, GLKS 30181_06, GLKS 30156_16, GLKS 30177_15, GLKS 30160_13, GLKS 30160_15 and GLKS 30177_20 of *S. chacoense*. This indicates that these genotypes need considerably less N to produce the same amount of biomass. A high NutE is often related to a good translocation of N from the root to the shoot and/or a reallocation from older leaves to the younger leaves in order to maintain the photosynthetic activity and eventually to the reproductive organs [8,36]. The superior performance of *S. chacoense* genotypes was also found in field studies where, amongst 39 wild potato accessions, genotypes of *S. microdontum* and *S. chacoense* revealed the highest total biomass (tubers + roots + shoots + fruits), a high NupE and N recovery from soil, even exceeding the performance of the control varieties cv. Russet Norkotah and cv. Red Norland [22]. The authors attributed the higher N recovery by the wild species to the deeper penetrating, denser, and more branched root system that is advantageous for nutrient uptake. However, it generally has to be considered that wild potato species form only small tubers, in some cases, produce stolons rather than tubers under long day conditions. Whether the high N recovery rate of the wild type will be maintained after crossing to cultivars needs detailed examination. Hybrids of *S. chacoense* and a haploid *S. tuberosum* line (USW551) were studied in the field with high and zero N supply by Errebhi et al. [23]. Here, hybrids showed highest N use efficiency and produced a total biomass (tubers + roots/stolons + shoots + fruits) higher or similar than that of commercial varieties, but tuber yield was low [23].

For a final assessment of the overall performance of the different genotypes under N deficiency, we studied the two stress indices SSI and STI based on the total DM. Zhao et al. [38] studied different indices to evaluate low N tolerance in maize, and advised to use several indices and not to rely on only one. The SSI for example, proposed by Fischer and Maurer [39] for evaluating the yield stability under stressed and non-stressed environments, does not consider the yield of a respective genotype in relation to the other genotypes tested under control conditions [38]. Cv. Kiebitz and GLKS 31559_14, for example, exhibited the lowest SSI and could thus be considered as the ones with the lowest N stress susceptibility. That is confirmed by no significant changes in shoot or root mass under low N as compared to high N. However, these genotypes produced a low total biomass during the three-week in vitro culture compared to the other genotypes tested even in the high N treatment. This might indicate that genotypes with a slow biomass development, and by this a probably rather low internal N demand, react less sensitive to a reduction in N supply than fast growing types with a strong biomass development. By calculating the STI, these genotype-specific growth rates were considered, and here genotypes with a high biomass development under both control and stress conditions rank best. Under this premise, cv. Kiebitz and GLKS 31559_14 were rather intolerant to N stress, while GLKS 30177_20, GLKS_30160_15, GLKS_30160_13 and GLKS 30181_06, GLKS_30177_15 and cv. Tomba were more tolerant. Finally, genotypes being among the best under both indices will be very interesting candidates for further research and pre-breeding. Here, we consider GLKS 30177_20, GLKS 30177_15 and GLKS 30160_15 of *S. chacoense* as the most relevant genotypes, because they combine high shoot and root biomasses in both treatments with a moderate reduction in shoot and root biomass under low N supply. Furthermore, the best performers revealed the highest share of root biomass in relation to total biomass, maintained root-DM:shoot-DM ratio under low N, but partitioned more N to the roots than other genotypes and revealed a high internal N utilization efficiency. *S. chacoense* is a well-known source of pest and disease resistance, resistance to cold-induced sweetening and abiotic stresses such as drought tolerance, but its tubers contain high levels of toxic steroidal glycoalkaloids [40,41]. As a diploid species (2n = 2x = 24, EBN 2), hybridization with tetraploid *S. tuberosum* (2n = 4x = 48, EBN 4) is possible after a ploidy reduction in the *S. tuberosum* parent to the diploid level, followed by backcrossing [42], but it is also an interesting future candidate for diploid breeding programs [43].

Apart from a sole comparison of different genotypes, our study highlights the variation of N use efficiency between genotypes within one population. The five genotypes of GLKS 30177 reacted differently to N stress. While GLKS 30177_15 and GLKS 30177_20 belong to the best performing genotypes, GLKS 30177_02 and GLKS 30177_17 exhibited a medium to low shoot DM, a strong reduction in root DM and root-DM:shoot-DM ratio under low N, low NutE and a low stability under stress (SSI). Instead, GLKS 30177_01 can be considered as an intermediate type. These results underline the high diversity of the different genotypes within a wild potato accession which is maintained as a population in gene banks. Furthermore, it highlights the importance to study, describe and maintain individual wild potato genotypes in order to promote the use of wild potato germplasm in breeding and research [44]. Therefore, the tested genotypes in this study are maintained clonally via in vitro propagation at the Gross Luesewitz Potato Collections.

Genotypes GLKS 34995_18, GLKS 31559_14 and GLKS 31559_11 of *S. tuberosum* subsp. *andigena* and *S. stenotomum*, belonging to the cultivated part of series Tuberosa and being most related to *S. tuberosum* according to the taxonomy of Hawkes [45], showed no outstanding performance in the respective experiment. Tuber starch contents as well as shoot and root biomass or N uptakes and efficiencies were on an intermediate to low level. Although revealing the highest tuber starch content, the genotype of *S. pinnatisectum* (GLKS 31600_10) showed a rather weak performance under the given experimental conditions. This was indicated by the lowest shoot and root biomass as well as the lowest N uptakes compared to the other wild potato genotypes. Since this was the sole genotype of this species, we can only speculate whether this is a generally low-yielding species or if the experimental conditions were unfavorable. However, its reactions to N deficit clearly distinguished it from the other genotypes. Although not statistically significant, absolute root mass increased by about 35% under N deficit and the proportion of root biomass in relation to total biomass increased most at the expense of the shoot mass. In relation to its exceptionally high tuber starch contents, it is worth studying the root parameters of this species in further experiments.

## 4. Materials and Methods

### 4.1. Plant Material

In total, a set of 28 accessions was selected from the Gross Luesewitz Potato Collections (GLKS, Gross Luesewitz, Germany) of the Leibniz Institute of Plant Genetics and Crop Plant Research (Table 6). The set comprised 15 accessions of the species *S. chacoense* Bitter, five accessions of *S. pinnatisectum* Dunal, and one each of *S. tuberosum* subsp. *andigena* Hawkes, *S. commersonii* Dunal, *S. hondelmannii* Hawkes and Hjerting, *S. jamesii* Torrey, *S. microdontum* Bitter, *S. sparsipilum* (Bitt.) Juz. and Bukasov, *S. stenotomum* Juz. and Bukasov, and *S. tarijense* Hawkes (Table 6). These species were selected because they are known for other interesting traits such as disease resistance and/or because they originate from regions with high temperature and low rainfall, and by this may additionally provide tolerance to heat and drought. The latter is especially expected from *S. chacoense*, which originates from the Chaco-Region, a hot and semi-arid region in southern America. Detailed passport data of the wild potato accessions maintained at the IPK Potato Collections in Gross Luesewitz are also available via the genebank information system (GBIS). For in vitro establishment, 50 seeds of the respective accession were pretreated in gibberellic acid (500 ppm) for 24 h at room temperature to improve germination. After that, seeds were treated with 5% NaClO solution to sterilize them and placed in a test tube (one seed per tube) containing about 6.0 mL of a solid culture medium under sterile conditions. The solid culture medium was composed as described by Murashige and Skoog [46]. The seeds were then placed in a climate chamber at 20 °C and 12 h of light (150–250 µmol m^−2^ s^−1^). After approximately four weeks, 20 well developed genotypes per accession were chosen for further experiments and multiplied. For multiplication, the plantlet of a respective genotype was cut in up to four nodal sections which were then transferred to new tubes with solid culture medium and grown in a climate chamber as described above. Prior to their cultivation in the respective experiments, genotypes were tested for quarantine diseases like virus (X, Y, L, S, M, A), potato spindle tuber viroid (PSTVd), bacteria (*Clavibacter michiganensis* ssp. *sepedonicus*, *Ralstonia solanacearum*) and Andean viruses (Andean Potato Latent Virus (APLV-Col, APLV-Col 2, APLV-Hu), Andean Potato Mottle Virus (APMoV-B, APMoV-H), Potato Black Ringspot Virus (PBRSV), Aracacha Virus B, Oca strain (AVB-O), Potato Virus T (PVT), Potato Virus V (PVV), Potato Yellowing Virus (PYV)).

### 4.2. Evaluation of Tuber Starch Contents

In 2013, 20 genotypes of each accession and three plantlets per genotype were transferred into pots (16 × 16 cm, 16 cm deep) filled with a turf-based planting substrate (95% white turf, 5% sand, 1.5 kg NPK (14% N, 16% P_2_O_5_, 18% K_2_O, micro nutrients), Einheitserde GmbH, Uetersen, Germany) and cultivated in the greenhouse. One accession was finally represented by 60 pots (20 genotypes and three plants per genotype). Plants were irrigated daily with rain- or tap water according to their needs. After natural senescence (three to four months after planting), irrigation was stopped, the aboveground plant biomass was removed, and the tubers were harvested separately for each genotype. Due to limited greenhouse capacities, starch content evaluations of accessions GLKS 30211, GLKS 30475, GLKS 31583, GLKS 31595 and GLKS 31559 were performed in 2014 in the same way as described above. Accordingly, accessions GLKS 31559, GLKS 32852 and GLKS 34995 were repeated in 2014 because too many genotypes were lost during greenhouse cultivation or too few tubers were produced. Furthermore, tubers of genotypes with high starch contents were re-cultivated in 2014 in order to validate the results.

### 4.3. Plant Material and Experimental Setup of N Experiments

For the N efficiency studies, genotypes were selected which had a tuber starch content higher than 28% of FM in 2013 and grew reliably in vitro and in the greenhouse. These comprised 16 genotypes from nine different accessions of *S. chacoense* and one genotype of *S. pinnatisectum*. Additionally, to cover a broader spectrum of *Solanum* species, two genotypes of *S. microdontum* and *S. stenotomum* as well as one genotype of *S. tuberosum* subsp. *andigena* were added (see Table 2). The selected genotypes were multiplied in vitro as described above. Finally, after having produced 40 plantlets per selected genotype, shoots tips of approximately 1.5 to 2.0 cm length were transferred to the testing system.

An in vitro method for early evaluation of nitrogen use efficiency traits as described in Schum et al. [17] was applied. In brief, 500 mL glass cultivation vessels were filled with 62 mL of a nutrient solution based on Murashige and Skoog [46] (Table 7). For the N experiments, two N levels were applied, containing 0.420 g L^−1^ N (control) and 0.105 g L^−1^ N (reduced N), respectively, being equivalent to 30 mmol L^−1^ and 7.5 mmol L^−1^ N. Ten shoot tips of one genotype were cultivated in one vessel for 21 days, and fixed via a perforated stainless steel plate. The transfer of the shoots to the experimental system was carried out under sterile conditions and the vessels were closed with a cellulose ring to enable gas exchange plus a glass lid to prevent contamination. They were placed in a climate chamber with 12 h of light and a constant temperature of 20 °C in a complete randomized design. All treatment × genotype combinations were repeated four times. Due to the high number of accessions and genotypes to be multiplied and tested, combined with the unequal growth rate of the different accessions, several consecutive experiments were conducted. For comparison, four modern varieties (cultivars, cv.) were used, kindly provided by the breeders; cv. Kiebitz (Norika, Germany), cv. Maxi (Bayerische Pflanzenzuchtgesellschaft, Germany), cv. Eurobravo and Tomba (Europlant Pflanzenzucht, Germany).

### 4.4. Laboratory Analyses and Calculations

#### 4.4.1. Tuber Starch Contents

Ten tubers of similar size (approx. 2 cm long) were selected per genotype, washed and analyzed immediately for their starch content via the underwater weight method using a balance (KERN PES 6200-2M, Kern & Sohn GmbH, Balingen, Germany) equipped with a cage to sink the tubers in water. The specific gravity (SG) was calculated based on the weight in air divided by difference of weight in air and weight under water. The starch content was calculated based on studies by Lunden [47] and as described in Meise et al. [20]:

Starch (% of FM)=−211.89+209.06* SG

#### 4.4.2. Yield, Nitrogen Uptake and Stress Indices

Harvested shoots and roots formed per in vitro vessel were weighed to determine fresh mass (FM). Dry mass (DM) was determined after drying the shoot and root biomass in an oven at 60 °C and weighing. The dry plant material was then ground in a mixer mill (Retsch, Tissue Lyser, Quiagen GmbH, Duesseldorf, Germany) for one minute at a frequency of 30 s^−1^ using 3 mm steel beads. After that, the dried and ground plant material was analyzed for its N content using an elementar analyzer (Eurovector EA 3000 b, HEKAtech GmbH, Wegberg, Germany). Shoot and root N uptake were calculated by multiplying the measured N content with the shoot or root dry mass. Total N uptake was calculated by summing up shoot and root N uptake. The root-DM:shoot-DM ratio was calculated by dividing the root DM by the shoot DM. Similarly, the N uptake into the root was divided by the shoot N uptake to reflect the partitioning of N in the plant, and was denoted as root-N:shoot-N ratio. The N uptake efficiency (NupE, %) was calculated by dividing the total N uptake by the amount of N supplied in the respective treatment:

$$\mathrm{NupE}\;\left(\%\right)=\frac{\mathrm{total}\;N\;\mathrm{uptake}\;\left(\mathrm{mg}\;\mathrm{per}\;\mathrm{vessel}\right)}{\mathrm{total}\;N\;\mathrm{supplied}\;\left(\mathrm{mg}\;\mathrm{per}\;\mathrm{vessel}\right)}$$

The relation of the total N taken up by the plant to the total dry mass produced is denoted as N utilization efficiency (NutE) and was calculated as follows:

$$\mathrm{NutE}\;\left(\mathrm{arb}.\;u.\right)=\frac{\mathrm{total}\;\mathrm{dry}\;\mathrm{mass}\;\left(\mathrm{mg}\;\mathrm{per}\;\mathrm{vessel}\right)}{\mathrm{total}\;N\;\mathrm{uptake}\;\left(\mathrm{mg}\;\mathrm{per}\;\mathrm{vessel}\right)}$$

Furthermore, the stress susceptibility index (SSI) was calculated based on total dry mass (SSI_DM_) as introduced by Fischer and Maurer [39]:

$$\mathrm{SSI}=\frac{\left(1-\mathrm{Ps}/\mathrm{Pc}\right)}{\left(1-\mathrm{meanPs}/\mathrm{meanPc}\right)}$$

where Ps is the parameter (DM, N uptake) determined under stress conditions and Pc is the parameter determined under control conditions. This is related to the mean of all genotypes tested under stress conditions (mean Ps) divided by the mean of all genotypes under control conditions (mean Pc). Additionally, the stress tolerance index was calculated as proposed by Fernandez [48]:

$$\mathrm{STI}=\frac{\mathrm{Pc}*\mathrm{Ps}}{\left(\mathrm{meanPc}\right)²}$$

### 4.5. Statistical Analyses

All statistical analyses were performed using the software R (version 3.3.2, R Foundation for Statistical Computing, Vienna, Austria) [49]. For tuber starch content, one-way analysis of variance (ANOVA) was applied to test for within- and between-accession variations. Finally, mean, minimum and maximum values as well as coefficient of variation (CV) were calculated for each accession. Pearson correlation coefficient was calculated using the RcmdrMisc package [50].

For dry mass and N uptake traits, two-way ANOVA was used to test the effect of genotype and treatment as well as their interaction on the respective trait. Because the climate chamber allows the cultivation of the plantlets under highly controlled and standardized conditions, all consecutive experiments were analyzed together in one model. A linear model using the “lmer” procedure of the package “lme4” [51] was applied, with genotype, treatment and genotype × treatment as fixed effects. Assumptions such as normality of residuals and homogeneity of variances were tested prior to ANOVA using q-q-plots, the Shapiro–Wilk normality test and the Levene’s test (package “cars” [52]). If assumptions were not met, data were log transformed. If significant factor effects were identified (*p* ≤ 0.05), post hoc comparison of means was performed using the Tukey test of the “lsmeans” package [53] to compare all means. In the results, significant differences between the control and reduced N are indicated by asterisks. The Dunnett’s test against the control of the package “multcomp” [54] was used to identify means differing significantly from the best cultivar. In the results, means significantly different (higher or lower) from cv. Tomba are indicated by the lowercase letter “b”. Additionally, means significantly higher than cv. Tomba are underlined.

The original data of the experiments are provided at the e!DAL repository [55] under the DOI 10.5447/ipk/2020/19.

## 5. Conclusions

We assessed the tuber starch content of 506 wild potato genotypes under greenhouse conditions. Of them, 50 revealed tuber starch contents above 28% of FM, clearly exceeding the starch contents of commercial cultivars. Amongst the wild types with high starch content, three were superior in terms of N utilization efficiency (NutE) as indicated by the in vitro screening in a climate chamber under high and low N levels for 21 days. GLKS 30177_15, GLKS 30177_20 and GLKS 30160_15 of species *S. chacoense* produced the highest shoot and root biomass under N stress and showed only a moderate reduction of the total biomass under low N compared to the high N treatment. NutE of these genotypes was high and exceeded that of most other wild types and the standard varieties. Combining two common stress tolerance indices (SSI and STI) proved to be a helpful tool for the identification of genotypes with a high and stable biomass production under stress compared to non-stress conditions. Based on our study, the identified genotypes of *S. chacoense* are a promising source for further research projects aiming to improve starch contents and N use efficiency in cultivated potato. Most wild potato genotypes of this study are maintained in vitro and are available at the IPK Gross Luesewitz Potato Collections.

## Figures and Tables

**Table 1:** Wild potato accessions of the Gross Luesewitz Potato Collections (GLKS) used in the study ranked according to their tuber starch content. Given are the number of genotypes tested per accession, the mean starch content (%) in the tubers, its minimum and maximum values and the coefficient of variation (CV %).

Accession GLKS	*Solanum*- Species	No. Genotypes	Starch Content (% of FM)			
			Mean	Min	Max	CV %
30211	*S. commersonii*	13	30.0	26.4	33.3	8.60
30916	*S. chacoense*	20	26.8	19.5	31.6	11.5
31595	*S. pinnatisectum*	15	26.8	18.9	30.6	11.5
30475	*S. jamesii*	17	25.7	12.7	32.6	18.7
30177	*S. chacoense*	20	24.8	15.4	31.4	16.6
30159	*S. chacoense*	19	24.3	19.0	30.9	12.0
30154	*S. chacoense*	20	23.2	11.0	31.8	17.9
30160	*S. chacoense*	19	23.2	15.8	32.7	16.8
31600	*S. pinnatisectum*	18	23.2	13.0	36.6	24.7
30156	*S. chacoense*	20	23.1	15.3	29.1	17.6
30191	*S. chacoense*	20	22.5	15.6	28.2	15.7
30197	*S. chacoense*	19	22.5	17.9	28.0	13.8
30181	*S. chacoense*	20	22.3	15.4	29.8	19.9
30995	*S. chacoense*	18	22.1	15.6	29.0	17.1
31025	*S. chacoense*	20	21.6	18.6	25.9	11.7
31610	*S. pinnatisectum*	19	21.5	14.7	31.9	24.2
30665	*S. chacoense*	19	21.1	13.0	27.2	16.0
30135	*S. chacoense*	20	20.4	13.8	33.0	23.9
31602	*S. pinnatisectum*	16	20.1	12.6	26.2	19.6
30688	*S. microdontum*	20	20.0	14.1	26.2	16.6
30148	*S. chacoense*	18	19.7	11.0	25.9	23.2
31605	*S. pinnatisectum*	7	19.6	16.1	22.7	11.4
30134	*S. chacoense*	20	19.3	14.0	25.6	18.0
32852	*S. hondelmannii*	18	19.2	13.7	24.4	17.2
31583	*S. tarijense*	19	16.9	13.5	20.7	11.8
34995	*S. tuberosum* subsp. *andigena*	16	16.6	12.6	21.6	17.9
30944	*S. sparsipilum*	20	16.0	11.4	22.2	18.8
31559	*S. stenotomum*	16	14.2	7.1	22.4	23.0

**Table 2:** Selected accessions and genotypes of the Gross Luesewitz Potato Collections used for the N efficiency experiments as well as their respective tuber starch contents (%) in 2013 and 2014.

Accession	Genotype	*Solanum*- Species	Starch Content (% of FM)		
GLKS	No.		2013	2014	Mean
30135	05	*S. chacoense*	28.0	25.3	26.7
30135	19	*S. chacoense*	33.0	29.8	31.4
30154	09	*S. chacoense*	31.8	25.2	28.5
30156	16	*S. chacoense*	29.1	26.7	27.9
30159	05	*S. chacoense*	30.9	36.9	33.9
30160	13	*S. chacoense*	29.5	26.2	27.9
30160	15	*S. chacoense*	32.7	-	32.7
30177	01	*S. chacoense*	24.2	29.0	26.6
30177	02	*S. chacoense*	30.9	27.3	29.1
30177	15	*S. chacoense*	30.1	26.0	28.1
30177	17	*S. chacoense*	28.3	32.0	30.2
30177	20	*S. chacoense*	31.4	-	31.4
30181	06	*S. chacoense*	28.6	-	28.6
30181	18	*S. chacoense*	29.8	26.2	28.0
30688	04	*S. microdontum*	26.2	31.8	29.0
30688	12	*S. microdontum*	25.3	23.9	24.6
30916	08	*S. chacoense*	31.6	29.7	30.7
30995	18	*S. chacoense*	29.0	28.7	28.9
31559	11	*S. stenotomum*	15.2	11.4	13.3
31559	14	*S. stenotomum*	22.1	16.6	19.4
31600	10	*S. pinnatisectum*	36.6	31.1	33.9
34995	18	*S. tuberosum* subsp. *andigena*	18.0	22.6	20.3

**Table 3:** Evaluated traits and variance explained (%) by the factors genotype, treatment and interaction. Given are the sum of squares (SSQ) resulting from two-factor analysis of variance and F-test.

Trait	Total SSQ	Genotype			Treatment			Genotype × Treatment			Residuals	
		SSQ	%	Sign.	SSQ	%	Sign.	SSQ	%	Sign.	SSQ	%
DM shoot mg vessel^−1^	3,164,366	2,167,098	68.5	***	697,852	22.1	***	113,436	3.58	***	185,980	5.88
DM root mg vessel^−1^	1,229,096	777,076	62.9	***	260,060	21.1	***	134,179	10.9	***	63,202	5.12
N uptake shoot ^a^	77.24	3.03	3.93	***	71.54	92.6	***	1.15	1.49	***	1.51	1.95
N uptake root ^a^	66.63	17.72	28.0	***	40.00	62.8	***	3.40	5.33	***	2.54	3.99
N uptake total ^a^	68.96	2.93	4.25	***	64.08	92.9	***	0.87	1.26	***	1.08	1.56
NutE ^a^	43.57	9.20	21.1	***	32.56	74.7	***	0.79	1.82	***	1.01	2.32
Root-DM:Shoot-DM ratio ^a^	31.26	21.15	67.7	***	1.87	6.00	***	4.81	15.4	***	3.43	11.0
Root-N:Shoot-N ratio ^a^	30.69	18.29	59.6	***	4.56	14.9	***	4.15	13.5	***	3.69	12.0

^a^ log transformation of the data prior to ANOVA; SSQ = sum of squares; % = percent share of total sum of squares, Sign. = significance of F-test (* *p* ≤ 0.05; ** *p* ≤ 0.01; *** *p* ≤ 0.001).

**Table 4:** Mean dry mass and N uptake of shoots and roots (mg vessel^−1^) of different wild potato genotypes cultivated under optimal and reduced N supply for 21 days in a climate chamber. Results of ANOVA and post hoc comparison of means.

Genotype	*Solanum*- Species	Shoot DM			Root DM			Shoot N			Root N			N Uptake Total		
		Control	Reduced	*p*	Control	Reduced	*p*	Control	Reduced	*p*	Control	Reduced	*p*	Control	Reduced	*p*
30135_05	*S. chacoense*	552 a	462 a	**	135 b	51.7 b	***	19.5 b	7.58 a	***	3.01 b	0.85 b	***	22.5 b	8.48 b	***
30135_19	*S. chacoense*	604 a	411 a	***	177 b	78.0 b	***	24.6 a	6.79 b	***	5.43 a	1.40 b	***	30.1 a	8.20 b	***
30154_09	*S. chacoense*	496 a	431 a	*	304 a	143 a	***	20.0 b	6.57 b	***	6.54 b	2.21 a	***	26.5 a	8.78 b	***
30156_16	*S. chacoense*	575 a	459 a	***	240 a	159 a	***	23.8 a	6.47 b	***	4.61 a	2.20 a	***	28.5 a	8.67 b	***
30159_05	*S. chacoense*	528 a	371 b	***	192 b	133 a	***	20.7 b	5.72 b	***	4.86 a	2.22 a	***	25.6 b	7.95 b	***
30160_13	*S. chacoense*	600 a	506 a	**	337 b	151 a	***	19.3 b	6.30 b	***	7.52 b	2.40 a	***	26.8 a	8.73 b	***
30160_15	*S. chacoense*	652 b	513 a	***	277 a	204 b	***	24.4 a	6.31 b	***	6.86 b	3.11 b	***	31.3 a	9.43 a	***
30177_01	*S. chacoense*	436 b	311 b	***	250 a	153 a	***	19.7 b	5.07 b	***	5.91 b	2.45 a	***	25.7 b	7.58 b	***
30177_02	*S. chacoense*	495 a	297 b	***	157 b	62.5 b	***	22.2 a	5.99 b	***	6.17 b	2.43 a	***	28.3 a	8.44 b	***
30177_15	*S. chacoense*	620 a	485 a	***	226 a	203 b	ns	24.7 a	6.95 b	***	4.56 a	2.66 b	***	29.2 a	9.61 a	***
30177_17	*S. chacoense*	280 b	196 b	**	156 b	74.5 b	***	17.3 b	5.19 b	***	4.55 a	1.76 a	***	22.1 b	6.96 b	***
30177_20	*S. chacoense*	682 b	549 b	***	318 b	219 b	***	25.5 a	6.85 b	***	5.00 a	2.99 b	***	30.6 a	9.96 a	***
30181_06	*S. chacoense*	680 b	508 a	***	291 a	117 a	***	23.0 a	7.45 b	***	5.29 a	1.85 a	***	28.3 a	9.84 b	***
30181_18	*S. chacoense*	397 b	335 b	*	76.0 b	61.5 b	ns	18.8 b	6.99 b	***	3.43 a	1.35 b	***	22.3 b	8.35 b	***
30688_04	*S. microdontum*	491 b	377 b	***	128 b	118 a	ns	23.5 a	7.17 b	***	4.50 a	2.08 a	***	28.1 a	9.27 b	***
30688_12	*S. microdontum*	497 a	384 a	***	139 b	116 a	ns	24.2 a	6.58 b	***	4.07 a	2.05 a	***	28.2 a	8.64 b	***
30916_08	*S. chacoense*	536 a	403 a	***	160 b	104 b	**	22.8 a	5.80 b	***	3.65 a	1.83 a	***	26.4 a	7.64 b	***
30995_18	*S. chacoense*	458 b	306 b	***	103 b	51.5 b	**	22.5 a	5.84 b	***	2.87 b	1.04 b	***	25.4 b	6.90 b	***
31559_11	*S. stenotomum*	498 a	266 b	***	195 b	95.0 b	***	23.6 a	6.40 b	***	4.59 a	1.51 a	***	28.2 a	7.91 b	***
31559_14	*S. stenotomum*	408 b	323 b	**	77.3 b	89.7 b	ns	22.6 a	7.01 b	***	3.19 b	1.34 b	***	25.8 b	8.36 b	***
31600_10	*S. pinnatisectum*	214 b	156 b	*	30.0 b	40.7 b	ns	11.3 b	5.49 b	***	1.48 b	1.44 a	ns	12.8 b	6.93 b	***
34995_18	*S. tuberosum* subsp. *andigena*	403 b	313 b	**	173 b	118 a	**	23.1 a	6.56 b	***	5.37 a	1.79 a	***	28.5 a	8.39 b	***
Eurobravo	*S. tuberosum* subsp. *tuberosum*	452 b	400 a	ns	157 b	102 b	**	22.6 a	7.48 a	***	4.70 a	1.68 a	***	27.3 a	9.17 b	***
Kiebitz	*S. tuberosum* subsp. *tuberosum*	302 b	268 b	ns	66.0 b	50.2 b	ns	19.9 b	7.58 a	***	2.96 b	0.97 b	***	22.9 b	8.56 b	***
Maxi	*S. tuberosum* subsp. *tuberosum*	456 b	374 b	*	164 b	95.0 b	***	23.6 a	7.74 a	***	4.00 a	1.38 b	***	27.6 a	9.12 b	***
**Tomba**	*S. tuberosum* subsp. *tuberosum*	**567 a**	**452 a**	***	**270 a**	**153 a**	***	**27.1 a**	**9.34 a**	***	**4.31 a**	**1.91 a**	***	**31.4 a**	**11.3 a**	***
mean		496	381		185	115		21.9	6.66		4.59	1.88		26.6	8.56	

The letters within one column indicate whether there is a significant difference to the best cultivar Tomba (“b”) or not (“a”, Dunnett’s test *p* ≤ 0.05), underlined are mean values significantly higher than that of cv. Tomba; asterisks indicate a significant difference between the treatments within one genotype (pairwise comparisons, Tukey adjustment; *** *p* ≤ 0.001, ** *p* ≤ 0.01, * *p* ≤ 0.05, ns = not significant).

**Table 5:** Mean root-DM:shoot-DM ratio as well as partitioning of N taken up by the different wild potato genotypes (root-N:shoot-N ratio) cultivated under optimal and reduced N conditions in a climate chamber for 21 days. Results of ANOVA and post hoc comparison of means.

Genotype	*Solanum*- Species	Root-DM:Shoot-DM Ratio			Root-N:Shoot-N Ratio		
		Control	Reduced	*p*	Control	Reduced	*p*
30135_05	*S. chacoense*	0.25 b	0.10 b	***	0.15 a	0.11 b	*
30135_19	*S. chacoense*	0.29 b	0.19 b	***	0.22 a	0.21 a	ns
30154_09	*S. chacoense*	0.61 a	0.33 a	***	0.33 b	0.34 b	ns
30156_16	*S. chacoense*	0.42 a	0.35 a	ns	0.19 a	0.34 b	***
30159_05	*S. chacoense*	0.36 a	0.36 a	ns	0.23 b	0.39 b	***
30160_13	*S. chacoense*	0.55 a	0.30 a	***	0.39 b	0.38 b	ns
30160_15	*S. chacoense*	0.42 a	0.40 a	ns	0.28 b	0.49 b	***
30177_01	*S. chacoense*	0.57 a	0.48 b	ns	0.30 b	0.48 b	***
30177_02	*S. chacoense*	0.32 b	0.20 b	***	0.28 b	0.41 b	**
30177_15	*S. chacoense*	0.36 a	0.42 a	ns	0.19 a	0.38 b	***
30177_17	*S. chacoense*	0.56 a	0.37 a	***	0.26 b	0.34 b	*
30177_20	*S. chacoense*	0.46 a	0.40 a	ns	0.20 a	0.44 b	***
30181_06	*S. chacoense*	0.43 a	0.23 b	***	0.23 b	0.25 a	ns
30181_18	*S. chacoense*	0.18 b	0.18 b	ns	0.18 a	0.19 a	ns
30688_04	*S. microdontum*	0.26 b	0.31 a	ns	0.19 a	0.29 b	**
30688_12	*S. microdontum*	0.28 b	0.30 a	ns	0.17 a	0.31 b	***
30916_08	*S. chacoense*	0.30 b	0.26 a	ns	0.16 a	0.32 b	***
30995_18	*S. chacoense*	0.22 b	0.17 b	*	0.13 a	0.18 a	*
31559_11	*S. stenotomum*	0.39 a	0.36 a	ns	0.19 a	0.24 a	ns
31559_14	*S. stenotomum*	0.19 b	0.27 a	*	0.14 a	0.19 a	*
31600_10	*S. pinnatisectum*	0.14 b	0.26 a	***	0.13 a	0.26 a	***
34995_18	*S. tuberosum* subsp. *andigena*	0.43 a	0.38 a	ns	0.23 b	0.27 a	ns
Eurobravo	*S. tuberosum* subsp. *tuberosum*	0.35 a	0.26 a	*	0.21 a	0.22 a	ns
Kiebitz	*S. tuberosum* subsp. *tuberosum*	0.22 b	0.19 b	ns	0.15 a	0.13 b	ns
Maxi	*S. tuberosum* subsp. *tuberosum*	0.36 a	0.25 a	*	0.17 a	0.18 a	ns
**Tomba**	*S. tuberosum* subsp. *tuberosum*	**0.47 a**	**0.34 a**	*	**0.16 a**	**0.20 a**	*
mean		0.36	0.29		0.21	0.29	

The letters within one column indicate whether there is a significant difference to the best cultivar Tomba (“b”) or not (“a”, Dunnett’s test *p* ≤ 0.05), underlined are mean values significantly higher than that of cv. Tomba; asterisks indicate a significant difference between the treatments within one genotype (pairwise comparisons, Tukey adjustment; *** *p* ≤ 0.001, ** *p* ≤ 0.01, * *p* ≤ 0.05, ns = not significant).

**Table 6:** Overview of the accessions analyzed for starch content and nitrogen use efficiency as well as passport data of the accessions and taxonomic classification (according to Hawkes [45]).

GLKS-Accession	*Solanum*-Species, Ploidy, EBN ^	Series *	Origin °
30211	*S. commersonii* Dunal (2x, 1)	COM	URY
30475	*S. jamesii* Torrey (2x, 1)	PIN	USA
31595, 31600, 31602, 31605, 31610	*S. pinnatisectum* Dunal (2x, 1)	PIN	MEX
31559	*S. stenotomum* Juz. and Bukasov (2x, 2)	TUBc	BOL
34995	*S. tuberosum* subsp. *andigena* Hawkes (4x, 4)	TUBc	UNK
32852	*S. hondelmannii* Hawkes and Hjerting (2x, na)	TUBw	BOL
30688	*S. microdontum* Bitter (2x, 3x, 2)	TUBw	ARG
30944	*S. sparsipilum* (Bitt.) Juz. and Bukasov (2x, 2)	TUBw	BOL
30134, 30135, 30148, 30154, 30156, 30159, 30160, 30177, 30181, 30191, 30197, 30665, 30916, 30995, 31025	*S. chacoense* Bitter (2x, 2)	YNG	ARG
31583	*S. tarijense* Hawkes (2x, 2)	YNG	UNK

^ EBN = Endosperm Balance Number; * COM = Commersonia, PIN = Pinnatisecta, TUBc = Tuberosa cultivated, TUBw = Tuberosa wild, YNG = Yungasensa; ° ARG = Argentina, BOL = Bolivia, MEX = Mexico, URY = Uruguay, USA = United States of America, UNK = unknown.

**Table 7:** Composition of the nutrient solution used in the control and reduced N treatment of the N efficiency experiments.

Chemical	Unit	Control	Reduced	Nutrients	Control	Reduced
NH_4_NO_3_	g/L	0.825	0.206	N	0.420	0.105
KNO_3_	g/L	0.950	0.238	K	0.784	0.784
KCl	g/L	0.701	1.226	Cl	0.545	0.795
CaCl_2_ × 6H_2_O	g/L	0.655		Ca	0.120	
MgSO_4_ × 7H_2_O	g/L	0.370		Mg	0.036	
KH_2_PO_4_	g/L	0.170		P	0.039	
FeSO_4_ × 7H_2_O	g/L	0.028		Fe	0.006	
Na × EDTA	g/L	0.037				
MnSO_4_ × H_2_O	mg/L	17.10		Mn	5.558	
ZnSO_4_ × 7 H_2_O	mg/L	8.600		Zn	1.955	
H_3_BO_3_	mg/L	6.200		B	1.084	
CuSO_4_ × 5 H_2_O	mg/L	0.025		Cu	0.006	
CoCl_2_ × 6H_2_O	mg/L	0.025		Co	0.012	
Na_2_MoO_4_ × 2H_2_O	mg/L	0.250		Mo	0.119	
Organic stock sol.	mL/L	1.0		S	0.056	
Sucrose	g/L	30				
